# Ontario Veterinary College

**Published:** 1893-12

**Authors:** 


ONTARIO VETERINARY COLLEGE.

*March 24th.—Degree V. S. (Veterinary Surgeon.)*

| | |
|---|---|
| Francis Abele, Jr. | Boston, Mass. |
| A. M. Adams | Canal Dover, O. |
| Charles H. Adams | Stouffville, Ont. |
| William E. Adams | Stouffville, Ont. |
| James Airth | Port Elgin, Ont. |
| Albert E. Alexander | Strathroy, Ont. |
| L. A. Anderson | Cincinnati, O. |
| John Armstrong | Locust Hill, Ont. |
| Albert C. Baker | Courtland, N. Y |
| Robert Barnes | Poplar Hill, Ont. |
| William C. Barth | Leavenworth, Kan. |
| John M. Boyles | Mongolia, Ont. |
| James Beath | Columbus, Ont. |
| F. M. Bertron | Canisteo, N. Y. |
| Oscar J. Biehn | Coopersburg, Pa. |
| Frank M. Blatchford | New Hamburg, Ont. |
| Albert T. Bowman | Canton, O. |
| James L. Brooks | Fingal, Ont. |
| Edward W. Brumter | Wooster, O. |
| Albert Bryant | Lucan Ont. |
| Robert Finlay Campbell | Whitby, Ont. |
| Eugene M. Casey | New Milford, Pa. |
| Fred H. Cassels | Tacoma, Wash. |
| Robert Cassels | Wingham, Ont. |
| George R. Christian | Glenns Valley, Ind. |
| William F. Coleman | Toronto, Ont. |
| Robert E. Collins | Lovelton, Pa. |
| Henry L. B. Coote | Minnedosa, Man. |
| C. M. Culbertson | Durham, Ont. |
| William Davidson | Harriston, Ont. |
| William H. Derr | Wooster, O. |
| James D. Deyell | South Monaghan, Ont. |
| P. A. Dillahunt | Springfield, O. |
| Henry Domville | St. John, N. B. |
| Eugene E. Dooling | Syracuse, N. Y. |
| George W. Dunlap | Goodville, Pa. |
| Nelson D. R. Eakin | Toronto, Ont. |
| Edwin W. Emery | Maumee, O. |
| Fred Evans | Avoca, Neb. |

| | |
|---|---|
| Albert C. Ewart | Wyoming, Ont. |
| Harry V. Fenn | Colchester, Eng. |
| John A. Filsinger | Syracuse, N. Y. |
| Duncan Fisher | Grandin, N. Dakota |
| Michael J. Foran | Auburn, N. Y. |
| Charles B. Frederick | Freeburg, O. |
| Frank E Freeman | Belfast, Me. |
| George D. Gibson | Brandon, Man. |
| Horace M. Gohn | Thornhill, Ont. |
| William T. Graham | Door Village, Ind. |
| Frank C. Grayson | Paxton, Ill. |
| Philip Harrison | Bad Axe, Mich. |
| William Harrison | Bad Axe, Mich. |
| John Hatton | Glenboro, Man. |
| E. M. Herrington | Picton, Ont. |
| Charles J. Hinkley | Odebolt, Ia. |
| Charles W. Holley | Ralston, Pa. |
| E. J. Hopkins | Newboro, Ont. |
| Geo. Howell | Vernon, Ont. |
| Arthur M. Humphrey | Darien Centre, N. Y. |
| Edwin H. Humphrey | Remington, O. |
| Buoy R. Ilsley | Weymouth, Nova Scotia |
| Richard J. Jelly | Mossley, Ont. |
| Edward L. Kalb | Rochester, Minn. |
| Milton J. Kellam | Rainham Centre, Ont. |
| Daniel M. Kellogg | Reynoldsville, N. Y. |
| Henry F. Kennedy | West Union, Ia. |
| William H. Kerr | Urbano. O. |
| Joseph Kime, Jr. | Chatham, Ont. |
| Daniel R. Kohler | Kuttztown, Pa. |
| Karl H. Kolbe | Napoleon, O. |
| Virgil Lathrop | Albany, N. Y. |
| F. E. Lawton | Paris, Ill. |
| Charles E. Leist | Circleville, O. |
| John P. Lemon | Cresco, Ia. |
| Frank M. Linscott | Holton, Kan. |
| Edwin D. Longacre | Longacre, Pa. |
| Charles B. McAndless | Ilderton, Ont. |
| W. J. H. McBride | Amherstburg, Ont. |
| George McCluskey | Orangeville, Ont. |
| James McGillicuddy | Walford, Ont. |
| James F. McGregor | Delaware, Ont. |

| | |
|---|---|
| Alex. M. McKay - - - | Morewood, Ont. |
| Alex. H. McKellar - - - | Washburn, Ia. |
| Arthur McKercher - - - | Petrolia, Ont |
| Ellis McLain - - - | Nanticoke, Ont. |
| John A. McMaster - - - | Guelph, Ont. |
| Harry T. Madden - - - | Greenfield, Ill. |
| R. H. Madill - - - | Streetville, Ont. |
| D. M. Mahomey - - - - | Ladoga, Ind. |
| George G. Manser - - - | Linwood, Ont. |
| John H. Marbes - - - | Syracuse, N. Y. |
| Harry Delos Martin - - - | Buffalo, N. Y. |
| John William Mather - - | Rohrsburg, Pa. |
| W. H. B. Medd - - - - | Larchwood, Ia. |
| J. Harvey Mettlen - - - | Wakefield, Neb. |
| Chester Miller - - - | Ottumwa, Ia. |
| Albert W. Moore. - - - | Charlevoix, Mich. |
| L. G. Moore - - - - | Trenton, N. Y. |
| W. J. Morgan - - - | Kingston, Ont. |
| Bruce D. Munro - - - | Whitehall, Pa. |
| Thomas H. Murray - - | Medo, Minn. |
| Thomas W. Orme - - - | London, Ont. |
| James W. Orr - - - | New Hamburg, Ont. |
| Thomas Packwood - - - | Goderich, Ont. |
| Charles E. Parker - - - | Dexter, Mich. |
| John M. Pattison - - - | Bentonville, Ind. |
| Romanzo Perkins - - - | Hardys, N. Y. |
| Harry H. Post - - - | Melville, Ont. |
| Edwin L. Price - - - | Reynoldsburg, O. |
| C. K. Rhodes - - - | Broadway, Va. |
| H. A. Rose - - - | Selkirk, Ont. |
| Isaac A. Ruby - - - - | North Liberty, O. |
| William H. Salisbury - - | Phelps, N. Y. |
| Harry G. Sands - - - | Welliversville, Pa. |
| Daniel E. Seller - - - | Minden City, Mich. |
| Albert G. Shirley - - - | Watford, Ont. |
| Elvin E. Shoebottom - - | Lucknow, Ont. |
| G D Showalter - - - - | Broadway, Va. |
| Charles J. Sigmond - - | Minneapolis, Minn. |
| Septimus Marshall Smith - - | Moosomin, N. W. T. |
| Andrew Spence - - - | Emerson, Man. |
| Oliver K. Steers - - - - | Midland, Ont. |
| John P. Stovea - - - | Shady Grove, Pa. |

| | |
|---|---|
| Elmer E. Sweeley | Warrensville, Pa. |
| Thomas J. Sweet | Moredan, Man. |
| William B. Switzer | Williamson, N.Y. |
| H. Linwood Tower | North Adams, Mass. |
| John B. Turner | Butler, Ill. |
| Alexander S. Tweedley | Buffalo, N. Y. |
| Leunis Van Es | Columbus, Neb. |
| Oscar Verschelden | St. Mary's, Kan. |
| Jacob E. Walcott | Napoleon, O. |
| William H. Walcott | Napoleon, O. |
| Thomas Walden | Gore's Landing, Ont. |
| William Bert Washburn | Richfield Centre, O. |
| Thomas E. Watson | Nelson, Ont. |
| Henry J. Weld | Delaware, Ont. |
| George Howard Welliver | Bloomsburg, Pa. |
| T. Townsend Whitling | Island Lake, Minn. |
| Jacob Wilhelm | Shakespeare, Ont. |
| Maurice E. Wilson | Estherville, Ia. |
| E. A. Wootton | Wellman's Corners, Ont. |
| Edwin C. Yoder | Kutztown, Pa. |
| George J. Young | Marshalltown, Pa. |
| Charles C. Yule | Weston, O. |

